# The role of children in transmission of SARS-CoV-2: A rapid review

**DOI:** 10.7189/jogh.10.011101

**Published:** 2020-06

**Authors:** Xue Li, Wei Xu, Marshall Dozier, Yazhou He, Amir Kirolos, Evropi Theodoratou, on behalf of UNCOVER

**Affiliations:** 1 Centre for Global Health, Usher Institute, University of Edinburgh, Edinburgh, UK; 2 College of Medicine and Veterinary Medicine, University of Edinburgh, Edinburgh, UK; 3 Department of Clinical Infection, Microbiology & Immunology, Institute of Infection, Veterinary & Ecological Sciences, University of Liverpool, Liverpool, UK; 4 Cancer Research UK Edinburgh Centre, Medical Research Council Institute of Genetics and Molecular Medicine, University of Edinburgh, Edinburgh, UK

## Abstract

**Background:**

Understanding the role of children in the transmission of SARS-CoV-2 is urgently required given its policy implications in relation to the reopening of schools and intergenerational contacts.

**Methods:**

We conducted a rapid review of studies that investigated the role of children in the transmission of SARS-CoV-2. We synthesized evidence for four categories: 1) studies reporting documented cases of SARS-CoV-2 transmission by infected children; 2) studies presenting indirect evidence on the potential of SARS-CoV-2 transmission by (both symptomatic and asymptomatic) children; 3) studies reporting cluster outbreaks of COVID-19 in schools; 4) studies estimating the proportions of children infected by SARS-CoV-2, and reported results narratively.

**Results:**

A total of 16 unique studies were included for narrative synthesis. There is limited evidence detailing transmission of SARS-CoV-2 from infected children. We found two studies that reported a 3-month-old whose parents developed symptomatic COVID-19 seven days after caring for the infant and two children who may have contracted COVID-19 from the initial cases at a school in New South Wales. In addition, we identified six studies presenting indirect evidence on the potential for SARS-CoV-2 transmission by children, three of which found prolonged virus shedding in stools. There is little data on the transmission of SARS-CoV-2 in schools. We identified only two studies reporting outbreaks of COVID-19 in school settings and one case report of a child attending classes but not infecting any other pupils or staff. Lastly, we identified six studies estimating the proportion of children infected; data from population-based studies in Iceland, Italy, South Korea, Netherlands, California and a hospital-based study in the UK suggest children may be less likely to be infected.

**Conclusions:**

Preliminary results from population-based and school-based studies suggest that children may be less frequently infected or infect others, however current evidence is limited. Prolonged faecal shedding observed in studies highlights the potentially increased risk of faeco-oral transmission in children. Further seroprevalence studies (powered adequately for the paediatric population) are urgently required to establish whether children are in fact less likely to be infected compared to adults.

**Note:**

We plan to update this rapid review as new data becomes available. These updates are available at https://www.ed.ac.uk/usher/uncover/completed-uncover-reviews.

COVID-19, caused by severe acute respiratory syndrome coronavirus 2 (SARS-CoV-2), was declared a pandemic on 11 March 2020 by the World Health Organization. Children are less likely to develop severe disease from COVID-19 compared to adults, although the reasons for this remain unclear [1]. Despite the fewer number of cases reported in children, there are concerns about asymptomatic or mildly symptomatic paediatric cases going undetected and unknowingly transmitting SARS-CoV-2 in the community or schools [2,3]. Understanding the role of children in the transmission of SARS-CoV-2 is of global interest and is urgently required given its policy implications in relation to reopening schools and intergenerational contacts. This rapid review aims to synthesise the latest evidence in relation to the role of children in the transmission of SARS-CoV-2.

## METHODS

### Literature search and eligibility criteria

We searched PubMed, medRxiv and the WHO COVID-19 database on 30 April 2020 with entry date limits from late 2019 (please see search strategies in the Appendix S1 of the **Online Supplementary Document**), to identify studies that investigated transmission of SARS-CoV-2 in children (0-18 years) or in schools. We reviewed titles and abstracts and subsequently full texts to identify publications based on predefined inclusion and exclusion criteria. We hand-searched reference lists of the retrieved eligible publications to identify any additional relevant studies. In particular, we included 1) studies reporting documented COVID-19 cases transmitted by SARS-CoV-2 positive children; 2) studies presenting indirect evidence on the potential of SARS-CoV-2 transmission by (both symptomatic and asymptomatic) children; 3) studies reporting cluster outbreaks of COVID-19 in schools; 4) studies estimating the proportions of children infected by SARS-CoV-2. Conversely, we excluded studies investigating clinical features and/or treatment of paediatric COVID-19 cases without any information on transmission. We included articles in peer-reviewed journals and pre-prints and excluded comments, conference abstracts, and interviews. We restricted studies to those reported in English or Chinese. In addition, we summarized and checked the references of previous reviews and policy briefs on the transmission of SARS-CoV-2 among children.

### Data extraction and evidence synthesis

Data relevant to the evidence for transmission of SARS-CoV-2 by children were extracted by four reviewers (XL, WX, YH, AK) and checked by a senior epidemiologist (ET). We synthesized evidence thematically and reported results narratively.

## RESULTS

**Figure 1:**
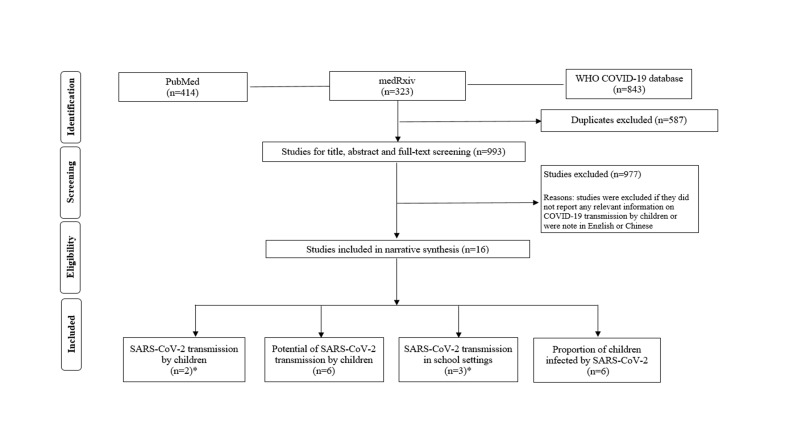
Flowchart summarizing study identification and selection. *One study reported documented cases of SARS-CoV-2 transmission by diagnosed children in school settings.

A total of 993 publications were retrieved and 16 unique studies were finally included for narrative synthesis (**Figure 1**): two studies investigating SARS-CoV-2 transmission from SARS-CoV-2 positive children [4,5]; six studies presenting indirect evidence on the potential of SARS-CoV-2 transmission by children [3,6-10]; two studies exploring school outbreaks [5,11] and one paediatric case report of tracing close contacts in schools [12]. We also found five studies [13-17], that addressed a relevant research question (ie, proportion of children infected as identified through random or targeted population testing) and we also report results from a large hospital-based study in the UK [18].

### Documented cases of SARS-CoV-2 transmission by children

We did not identify any studies directly addressing the transmission dynamics of SARS-CoV-2 by children. There is limited evidence detailing transmission of SARS-CoV-2 from children (**Table 1**).

**Table 1:** Characteristics of studies with documented cases of COVID-19 transmission by children

Author, country, study design	Population setting, patient demographics, clinical characteristics	Primary results
**Cai** [4], China (Shanghai), Case series (DOI: 10.1093/cid/ciaa198)	**Population setting:** Ten patients admitted to a children’s hospital for screening based on presentation with acute fever and or respiratory symptoms AND an epidemiological link to an adult case/exposure to an epidemic area.	**Confirmed transmission:** Transmission from infected infant to adults: n = 2. 3-mo-old infant whose two parents developed symptomatic COVID-19 seven days after looking after the infant. Source of infant infection not reported. Infant had positive nasopharyngeal swabs for 8 d.
	**Demographics:** Age: 3-131 months (mean: 74 months). Gender: male n = 4, female n = 6.	
	**Clinical characteristics:** Presentation: Fever n = 8 (80%); cough n = 6 (60%); sore throat n = 4 (40%); stuffy nose n = 3 (30%); sneezing and rhinorrhoea n = 2 (20%). RNA positive within 4-48 h after symptom onset. RNA (nasopharyngeal/throat swabs) undetectable within 6-22 days (mean: 12 d) after illness onset.	**Mean time to transmission/symptoms onset:** Not reported. Parents developed symptomatic COVID-19 seven days after looking after the infant.
**National Centre for Immunisation Research and Surveillance (NCIRS)** [5]. Australia (New South Wales). School-based study. Available at: http://ncirs.org.au/sites/default/files/2020-04/NCIRS%20NSW%20Schools%20COVID_Summary_FINAL%20public_26%20April%202020.pdf	**Population setting:** 15 schools (10 high school and 5 primary schools); a total of 18 COVID-19 cases (9 students and 9 staff) 18 individuals (9 students and 9 staff) from 15 schools were confirmed as COVID-19 cases; 735 students and 128 staff were close contacts of these initial cases.	**Confirmed transmission:** Transmission from infected students/staff to students: n = 2. No confirmed transmission from students to staff. One secondary case (child in a high school) was presumed to have been infected following close contact with two student cases. The other secondary case (child in a primary school) was presumed to have been infected by a staff member (teacher) who was a case.
	**Demographics**: Not reported	**Mean time to transmission/symptoms onset:** One of them was diagnosed by nose/throat swab testing 5-10 days after the last contact and one had a positive antibody test 4 weeks after their exposure.
	**Clinical characteristics**: Not reported	

A case series from China described 10 paediatric patients admitted to a children’s hospital in China [4]. They identified one potential case of SARS-CoV-2 transmission from an infant to parents, who subsequently developed symptomatic COVID-19 seven days after looking after the infant. This is probably the first piece of direct evidence indicating children as a source of adult infection.

A school-based study (10 high school and five primary schools) from New South Wales traced the close contacts (735 students and 128 staff) of 18 initial COVID-19 cases (nine students and nine staff) to see who they subsequently infected. They found that two children (secondary cases) may have contracted COVID-19 from the initial cases at their schools [5]. The authors highly suspected, but were unable to confirm, that one of the pupils was infected after close contact with two pupils in high school. They found no evidence of children infecting teachers or staff members.

### Indirect evidence of the potential for SARS-CoV-2 transmission by children

We identified six studies [3,6-10] presenting indirect evidence of the potential of SARS-CoV-2 transmission by children (**Table 2**).

**Table 2:** Characteristics of studies reporting indirect evidence of the potential for COVID-19 transmission by children

**Yung** [6]. Singapore (Infectious Disease Service, KK Women's and Children's Hospital). Case report (DOI: 10.7326/M20-0942)	**Population setting:** A 6 month-old infant was admitted for isolation because both parents tested positive for COVID-19. On admission, infant was asymptomatic, but nasopharyngeal swabs confirmed COVID-19 infection with very high viral load.	**Confirmed transmission:** On day 2 of admission, the infant's isolation environment and the personal protective equipment (PPE) of a health care worker (HCW) who was looking after the infant were sampled. For the infant, Ct values on real-time PCR for the N gene and Orf1ab gene were 18.8 and 18.6, respectively, while urine and stool samples remained negative. For the isolation environment, the infant's bedding, the cot rail, and a table situated 1 m away (all 3 environment samples) were found to be positive for SARS-CoV-2. The SARS-CoV-2 RdRp gene Ct values for the bedding, cot, and table were 28.7, 33.3, and 29.7, respectively. For all 3 samples from the HCW’s, PPE were found to be negative for SARS-CoV-2.
	**Demographics:** Age: 6 months. Gender: NA	In summary, a generally well infant with COVID-19 can contaminate the environment with PCR-detectable virus. Despite close physical contact with the infant during feeding, the evidence of SARS-CoV-2 on the gown of the HCW was not found.
	**Clinical characteristics:** The cycle threshold (Ct) values for N gene and Orf1ab gene polymerase chain reaction (PCR) assay were 15.6 and 13.7, respectively, on the day of admission.	
**Xu** [7]. China (Guangzhou, Guangdong Province). Single-center prospective observational study (DOI: 10.1038/s41591-020-0817-4)	**Population setting:** Between 22 January 2020 and 20 February 2020, 745 ‘highly suspected’ children were screened by real-time RT–PCR using nasopharyngeal swabs to detect people with SARS-CoV-2 infection. 10 children tested positive and were admitted to Guangzhou Women and Children’s Medical Centre.	**Transmission route:** Four of them had definite contact history with a confirmed patient, seven were from families with a cluster of infection and 7 had travel history to epidemic areas in Hubei Province 2 weeks before the onset of infection.
	**Demographics:** Age: 2-188 months (mean: 90.5 months). Gender: male n = 6, female n = 4	**Faecal viral shedding:** Positive real-time RT–PCR results in rectal swabs in 8 out of 10 paediatric patients, which remained detectable well after nasopharyngeal swabs turned negative, suggesting that the gastrointestinal tract may shed virus and faeco–oral transmission may be possible.
	**Clinical characteristics:** Presentation: fever n = 7 (70%); cough n = 5 (50%); sore throat n = 4 (40%); rhinorrhoea n = 2 (20%); diarrhoea n = 3 (30%); more than one sign n = 6 (60%)	
**Ma** [8]. China (Jinan, Shangdong Province), case series (DOI: 10.1016/j.jmii.2020.03.010)	**Population setting:** The study found 8 of 27 (29.6%) patients, all of whom were diagnosed with mild to moderate infection and discharged 1–2 weeks ago, showed positive PCR results in their stool but negative results in their respiratory specimens. Six (75%) of these 8 patients were children.	**Transmission route:** All 6 children had close contact with infected family members.
	**Demographics:** Age: 11-108 months (mean: 55.8 months). Gender: male n = 2, female n = 4	**Faecal viral shedding:** SARS-CoV-2 can be shed in the stool of patients in the recovery phase. Children show a longer shedding time than adults.
	**Clinical characteristics:** Presentation: fever n = 2 (33.3%)	
**Xing** [9]. China (Qingdao, Shandong Province), case series (DOI: 10.1016/j.jmii.2020.03.021)	**Population setting:** From January 17, 2020 to February 23, 2020, three paediatric cases of COVID-19 were reported in Qingdao, Shandong Province, China. Epidemiological, clinical, laboratory, and radiological characteristics and treatment data were collected. Patients were followed up to March 10, 2020, and dynamic profiles of nucleic acid testing results in throat swabs and faecal specimens were closely monitored.	**Transmission route:** None of these children had travel history outside of Qingdao one month before onset of the disease. All three children were infected because of close contact with infected family members. There was no evidence showing the virus was transmitted from the children to others.
	**Demographics:** Case 1: age: 1.5 years; gender: male. Case 2: age: 5years; gender: male. Case 3: age: 6years; gender: female	**Faecal viral shedding:** Case 1 and 2: RT-PCR results remained positive in stools of the two children for 8 and 20 d, respectively, after nucleic acid turning negative in respiratory samples. Case 3: Clearance of SARS-CoV-2 in stool samples occurred 20 days after viral RNA in respiratory specimens turning negative.
	**Clinical characteristics:** Presentation: fever n = 3 (100%); cough n = 1 (33.3%); stuffy nose n = 1 (33.3%); abdominal pain n = 1 (33.3%); diarrhea n = 1 (33.3%)	
**Terry** [10]. Germany (Berlin). Retrospective study	**Population setting:** From January to 26 April 2020, virology laboratories at Charité and Labor Berlin screened 59 831 patients for COVID-19 infection, 3712 (6.2%) with a positive real-time RT-PCR result. Patients were divided according to two categorizations to investigate whether there is a relationship between patient age and viral load. The first categorization is based on ten-year brackets. The second categorization is based on broad social strata: kindergarten (ages 0-6), grade school (ages 7-11), high school (ages 12-19), university (ages 20-25), adult (26-45), and mature (age over 45).	**Viral load distribution:** Kindergarten (ages 0-6): n = 37 (mean:5.16; SD: 1.97); Grade school (ages 7-11): n = 16 (mean:5.36; SD: 2.21); High school (ages 12-19): n = 74 (mean:4.78; SD: 1.78); University (ages 20-25): n = 267 (mean:4.37; SD: 1.60); Adult (ages 26-4 5): n = 1247 (mean:5.23; SD: 1.87); Mature (age over 45): n = 2071 (mean:5.28; SD: 1.95). The study found no significant differences in viral load exists between different age subgroups. However, there were smaller sample sizes in the paediatric age groups.
	**Demographics:** Kindergarten (ages 0-6): n = 1759); Grade school (ages 7-11): n = 623; High school (ages 12-19): n = 1790; University (ages 20-25): n = 4587; Adult (ages 26-45): n = 23 665; Mature (age over 45): n = 27 407	
	**Clinical characteristics:** Positive PCR counts and percentages. Kindergarten (ages 0-6): n = 37 (2.10%); Grade school (ages 7-11): n = 16 (2.57%); High school (ages 12-19): n = 74 (4.13%); University (ages 20-25): n = 267 (5.82%); Adult (ages 26-45): n = 1247 (5.27%); Mature (age over 45): n = 2071 (7.56%)	
**Dong** [3]. China (nationwide), Retrospective study (DOI: 10.1542/peds.2020-0702)	**Population setting:** Nationwide case series of 2135 paediatric patients with COVID-19 reported to the Chinese Center for Disease Control and Prevention from January 16, 2020, to February 8, 2020, were included. There were 728 (34.1%) laboratory-confirmed cases and 1407 (65.9%) suspected cases.	**Transmission route:** Approximately half of the patients were from Hubei province (981; 46.0%), whereas 396 (18.5%) case patients were from Anhui, Henan, Hunan, Jiangxi, Shanxi and Chongqing, which border Hubei province. In the spatial distribution, there was a clear trend that disease spread rapidly from Hubei province to surrounding provinces and cities over time. There were more children infected in the areas around Hubei province than in areas farther away except for Heilongjiang province. This study provided strong evidence at the start of the outbreak of human-to-human transmission.
	**Demographics:** Age: 1 day–18 years (mean: 7 years); Gender: male n = 1208 (56.6%), female n = 927 (43.4%)	
	**Clinical characteristics:** Regarding the severity (including both confirmed and suspected cases), 94 (4.4%), 1088 (51.0%), and 826 (38.7%) cases were diagnosed as asymptomatic, mild, or moderate, respectively; accounting for 94.1% of all cases. However, the proportion of severe and critical cases was 10.6%, 7.3%, 4.2%, 4.1%, and 3.0% for the age groups, 1, 1 to 5, 6 to 10, 11 to 15, and over 15 years, respectively. These results suggest that young children, particularly infants, were vulnerable to SARS-CoV-2 infection.	

One study assessed environmental contamination in the isolation room of an infected infant [6]. Although this infant was asymptomatic, nasopharyngeal swabs confirmed COVID-19 infection with a very high viral load. The presence of SARS-CoV-2 was detected on the surfaces exposed to the infant (eg, bedding, cot, table), showing a downward gradient of viral load with increasing distance from the infant. The surfaces proximal to the infant were assumed to be contaminated through crying or drooling. Findings from this study indicate the potential risk of airborne transmission or transmission through indirect contact of fomites, even in well infants with PCR-detectable virus. This study also reaffirms the importance of hand hygiene when caring for infants with COVID-19.

At least three studies have shown that children show prolonged faecal shedding compared to adults (and in some cases longer than four weeks) [7-9]. Notably, SARS-CoV-2 remained detectable in stool samples well after nasopharyngeal swabs turned negative. The researchers of one study also found that the viral load of SARS-CoV-2 in the gastrointestinal tract was greater and lasted longer than that in the respiratory system [9]. These findings lead to concerns about the potential for faeco-oral transmission of SARS-CoV-2, particularly in infants and children who are not toilet-trained and who have poorer hand hygiene. A study of 3712 COVID-19 patients analysed the variance of viral loads in patients of different age categories [10]. They initially reported a similar viral load in children as in adults [10], however, re-analysis of these data show that young children (<10 years old) had statistically significant lower viral load [19].

It is estimated that the proportion of infected children with latent asymptomatic or with mild symptoms of respiratory illness is higher than in adults, which highlights the possibility that children and young adolescents may be potential sources of undetected community transmission [3]. A nationwide case-series of 2135 paediatric patients with COVID-19 reported to the Chinese Centre for Disease Control and Prevention found that more than 90% had asymptomatic (4%), mild (50.9%), or moderate (38.8%) symptoms [3]. This is a hospital-based study, which may underestimate the true rate of asymptomatic infection, since many asymptomatic children are unlikely to be hospitalised or tested [3]. While there are studies describing the infective potential of asymptomatic adult cases [20,21], few describe this in children.

### SARS-CoV-2 transmission in school settings

Specific evidence related to the transmission of SARS-CoV-2 in schools is lacking, which is probably due to the early closure of schools at the start of the pandemic in many countries. We identified only two studies reporting a cluster outbreak of COVID-19 in school settings [5,11] and one case report of a paediatric case attending school but not infecting any other pupils or staff [12] (**Table 3**).

**Table 3:** Characteristics of studies reporting COVID-19 transmission in school settings

**National Centre for Immunisation Research and Surveillance (NCIRS)** [5]. Australia (New South Wales). School-based study. Available at: http://ncirs.org.au/sites/default/files/202004/NCIRS%20NSW%20Schools%20COVID_Summary_FINAL%20public_26%20April%202020.pdf	**Population setting:** 15 schools (10 high school and 5 primary schools); a total of 18 COVID-19 cases (9 students and 9 staff) 18 individuals (9 students and 9 staff) from 15 schools were confirmed as COVID-19 cases; 735 students and 128 staff were close contacts of these initial cases.	**Confirmed transmission:** Transmission from infected students/staff to students: n = 2; No confirmed transmission from students to staff; One secondary case (child in a high school) was presumed to have been infected following close contact with two student cases. The other secondary case (child in a primary school) was presumed to have been infected by a staff member (teacher) who was a case.
	**Demographics**: Not reported	**Mean time to transmission/symptoms onset:** One of them was diagnosed by nose/throat swab testing 5-10 days after the last contact and one had a positive antibody test 4 weeks after their exposure.
	**Case confirmation:** Participants were swabbed for SARS-CoV-2 virus testing and had a blood sample taken to detect antibodies to the virus.	
**Arnaud** [11]. French (Oise). Retrospective closed cohort study (DOI: 10.1101/2020.04.18.20071134)	**Population setting:** 661 participants included pupils, their parents and siblings, as well as teachers and non-teaching staff of a high-school linked to a cluster outbreak of COVID-19.	**Confirmed transmission:** 92 out of 240 pupils had anti-SARS-CoV-2 antibodies, and the infection attack rate (IAR) was 38.3% among children. The overall infection attack rate (IAR) was 40.9% in the high school group, and was 10.9% in parents and siblings of the pupils.
	**Demographics:** Age: ≤14 years (n = 37); 15-17 years (n = 205)	
	**Case confirmation:** A participant with positive serology at the time of blood sampling was considered as a confirmed case.	
**DANIS** [12]. French (Alps), a case report (DOI: not available)	**Population setting:** A paediatric patient was confirmed as a secondary case by close contacts with the index case in France. 112 school contacts with this child case were identified from 3 different schools and 1 ski class.	**Confirmed transmission:** No confirmed transmission. The paediatric case attended three schools while symptomatic, but did not transmit the virus.
	**Demographics:** 9 year-old child with respiratory symptoms	
	**Case confirmation:** SARS-CoV-2 virus was tested by the RT-PCR from either nasopharyngeal swabs or endotracheal aspirates in any of these school contacts monitored.	

One of these studies retrospectively followed up 661 pupils and staff in a French high school affected by an outbreak of COVID-19 [5]. They found that 40.9% of pupils and staff (with no significant difference between the two groups) became infected by school contacts. For household contacts, 10.9% of parents and siblings of infected pupils were also infected. However, it should be noted that almost all the students in the study were aged 15-17 years of age, and appeared to have similar disease characteristics to young adults. Therefore, the rate of infection reported might be not applicable to younger children.

A school-based study from New South Wales, identified 18 individuals (nine pupils and nine staff) from 15 schools (10 high schools and five primary schools) that were confirmed COVID-19 cases [11]. A total of 735 pupils and 128 staff were close contacts of these initial 18 cases and two secondary cases were reported. The first secondary case was a high-school student, who was presumed to have been infected following close contact with two student cases. The second secondary case was a primary school student, who was presumed to have been infected by a staff member (teacher). No teachers or staff members contracted COVID-19 from any of the initial school cases.

One case report describes a paediatric patient who was infected during a holiday in France [12]. Despite a large number of contacts in different classes after returning to the UK, the paediatric case did not transmit the disease to any other pupils.

### Proportion of children infected by SARS-CoV-2

We next sought to identify studies which estimated the proportion of children infected in the community. We included five studies [13-17] estimating the proportion of children infected through either random or targeted population testing, and a large hospital based study from the UK [18] (**Table 4**).

**Table 4:** Characteristics of studies reporting the proportions of children infected by COVID-19

**Gudbjartsson** [13]. Iceland, population-based screening (DOI: 10.1056/NEJMoa2006100)	**Population setting:** 9919 persons (564 children) from the high-risk population (mainly those who were symptomatic, had recently travelled to high-risk countries, or had contact with infected persons), and 13080 persons (848 children) from the population-screening programme	**Proportion of children infected by COVID-19:** 38 (6.7%) children from the high-risk group tested positive, in contrast to 1183 of 8635 persons (13.7%) who were 10 years of age or older. In the population-screening group, none of the 848 children tested positive
	**Demographics:** Age: <10 years	
	**Case confirmation:** RNA sequencing	
**Enrico** [14]. Italy (municipality of Vo’). Population-based survey (DOI: 10.1101/2020.04.17.20053157)	**Population setting:** 5155 persons (234 children) from 2 population-based surveys	**Proportion of children infected by COVID-19:** None of the 234 children tested positive.
	**Demographics:** Age: <10 years	
	**Case confirmation:** Real time RT-PCR	
**COVID-19 National Emergency Response Centre** [15]. Republic of Korea, population-based survey (DOI: 10.24171/j.phrp.2020.11.2.05)	**Population setting:** 7755 cases (480 children) from 2 population-based surveys	**Proportion of children infected by COVID-19:** 1% of confirmed cases were <10 years old and 5.2% were 10-19 years old.
	**Demographics:** Age: 0-9 years (n = 75); 10-19 years (n = 405)	
	**Case confirmation:** Laboratory-confirmed cases	
**National Institute for Public Health and Environment. Children and COVID-19 (PIENTER Corona study)** [16]. Netherlands, population-based survey (DOI: Not available)	**Population setting:** 6100 persons (480 children) from the PIENTER Corona study	**Proportion of children infected by COVID-19:** 2% tested positive for antibodies in their blood.
	**Demographics:** Age: <20 years	
	**Case confirmation:** Blood antibodies	
**Eran** [17]. U.S.A (Santa Clara, California), community-based survey (DOI: 10.1101/2020.04.14.20062463)	**Population setting:** 3330 persons (889 children) recruited from residents in Santa Clara county	**Proportion of children infected by COVID-19:** Among 621 children who were tested, 1.4% of children 0-4 years old and 1.5% of children 5-18 years old tested positive.
	**Demographics:** Age: <19 years	
	**Case confirmation:** Blood antibodies	
**Annemarie** [18]. UK (166 UK hospitals). Hospital-based prospective observational cohort study (DOI: 10.1101/2020.04.23.20076042)	**Population setting:** 16 749 people with COVID-19 diagnosed from 166 UK hospitals between 6 February and 18 April 2020.	**Proportion of children infected by COVID-19:** Severe COVID-19 infections are rare in those under 18 years of age, comprising 1.4% of those admitted to hospital. Only 0.8% of those in the study were under 5 years of age.
	**Demographics:** The median age was 72 years (IQR 57, 82; range 0, 104), the median duration of symptoms before admission was 4 days (IQR 1, 8) and the median duration of hospital stay was 7 days (IQR 4, 12).	

Targeted (n = 9199) and random population screening (n = 13 607) from Iceland found that children under 10 were less likely to test positive than those 10 and over (6.7% vs 13.7% for targeted testing; and 0% vs 0.8% for random population testing) [13]. The Italian principality of Vo tested >85% of their population following their first death from COVID-19 and found no positive cases in children despite 2.6% of the population testing positive [14]. A demographic breakdown of the first 7755 laboratory-confirmed cases in South Korea (where extensive community testing has been implemented) showed that only 1% of the confirmed cases were <10 years old and 5.2% were 10-19 years old [15]. A study in the Netherlands is undertaking community serology testing for antibodies against SARS-CoV-2 and in their first release of preliminary results they have found 4.2% of adults were positive compared to 2% of those aged <20 years [16]. Finally, a COVID-19 antibody seroprevalence study in Santa Clara County, California showed that positivity of the antibody test was not significantly different across age groups (but children were from the same households of the adults that were selected for testing) [18]. There is no population-based study in the UK to accurately assess the level of infection in the community. An ISARIC study of 16 749 hospitalised UK patients with COVID-19 from 166 UK hospitals found that only 2% of the patients were under 18 years old (n = 239 patients) and 1.1% were under 5 years old (n = 139 patients) [18].

## DISCUSSION

This rapid evidence review summarises the currently available evidence on the role of children in SARS-CoV-2 transmission. The key messages based on the findings of this review are: (i) Case series and outbreak reports detailing transmission in children are few. Those studies which are available demonstrate that transmission by children is possible but do not quantify the likelihood of transmission in children compared to adults. (ii) There is theoretical evidence that alternative transmission routes such as faeco-oral transmission may pose a higher risk in children. Further investigation is required to confirm whether longer viral shedding times in stools in children pose a higher risk of transmission. (iii) There is little evidence on transmission dynamics in school settings. Given the current paucity of data, further investigation and close monitoring will be essential where schools have re-opened and in settings where schools have remained open. (iv) Children are infected, but perhaps less frequently than adults. However, these studies are limited by small numbers and additional community seroprevalence surveys are needed to confirm the proportion of children infected.

Direct evidence showing children as a source of transmission is scarce and largely based on small studies or studies investigating few paediatric cases. We identified two studies indicating that children can transmit COVID-19 to other children or adults [4,5], however, these studies have a high degree of uncertainty. The early case series of paediatric cases in China [4] (providing evidence on children as a source of adult infection) has been questioned by the Swiss National Science Task Force group [22], who stated that a check of the original data does not confirm the reported transmission from the infant to their parents. For the other school-based study, the authors conclude that it is likely that a pupil was infected by one of two other pupils in a high school environment, but they are not certain due to the difficulty in tracking transmission chains in such a widespread outbreak [5].

While there is lack of direct evidence on the frequency that children transmit the disease, indirect evidence illustrates the potential risk of viral transmission by children. First, there is evidence showing that an infected infant contaminated (with SARS-CoV-2) the surfaces they were in contact with, thus rendering the risk of transmitting the virus to caregivers through indirect contact with fomites [6]. This finding reinforces the need for caregivers of children to practice hand hygiene as recommended by the WHO. Second, persistent shedding of SARS-CoV-2 in stools of infected children raises the possibility of faeco-oral transmission. This potential is evidenced by at least three studies, which demonstrated that SARS-CoV-2 may be present in the gastrointestinal tract for a longer duration than viral presence in the respiratory system [7-9]. Findings from these studies suggest that paediatric patients discharged on the basis of a negative respiratory test may remain a potential source of viral transmission and indicate that further precautions may need to be taken to avoid secondary infection during the convalescent phase. Third, several studies reporting that children are more likely to be asymptomatic or mild symptomatic carriers of SARS-CoV-2 lead to the concern that children may transmit the virus to other groups covertly [2,3]. The possibility of asymptomatic transmission of SARS-CoV-2 has been reported in adults by several studies [20, 21], while studies specifically targeting the infectiousness of asymptomatic children are yet to be carried out. Future studies aiming to understand children’s contribution to asymptomatic transmission of SARS-CoV-2 should be given priority, as this will influence the balance of control measures for identification and isolation of children and for tracing and quarantining close contacts.

Few studies have described SARS-CoV-2 transmission in schools. There are two studies that investigated school outbreaks of COVID-19 [5,11] and one case report of an infected child attending school without infecting other pupils or staff members [12]. Data from both virus and antibody testing suggest that there is limited evidence to suggest significant spread among children and from children to adults. This is consistent with studies showing the majority of infected children are related to family-cluster outbreaks and showing low rates of infection in children [3,23].

Understanding the proportion of SARS-CoV-2 infection in children will help to determine children’s relative contribution to SARS-CoV-2 transmission in the community. Based on the infection rates reported from Iceland, Italy, South Korea, Netherlands, and US [13-17], it seems increasingly likely that there are comparatively fewer children with COVID-19 in the community, particularly younger children under the age of 10. Data from these studies confirm that the percentage of children among the confirmed COVID-19 patients is small, varying from 1% in young children up to 6% in older children. It should be noted that these studies are limited by the small numbers of children tested and the potential of biased non-random sampling. Better seroprevalence studies are therefore needed to understand whether infection rates in the community are truly lower among children. The reason why COVID-19 seems to affect children less often and less severely than adults is not yet fully understood, but it could be partially explained by the relatively fewer opportunities for children to expose themselves to people beyond family members and school mates. Given the relatively small proportion of infections and lower chance of exposure, children are unlikely to have been significant drivers of the epidemic so far, and there is not any evidence yet to indicate they are at higher risk of causing super-spreading events in the community.

There is insufficient evidence to robustly answer the key study question. The absence of conclusive evidence on children’s role in transmission increases the uncertainty in relation to policy decisions on school closures and re-openings. During the COVID-19 pandemic, many countries have taken precautionary measures by closing schools to slow transmission among children. As the rate of infection subsides in some areas, some countries have started re-opening schools, and this further highlights the urgency of obtaining robust data on transmission in children and monitoring the impact re-opening schools have on country-level epidemics. A phased re-introduction of face-to-face learning in schools has started in Taiwan (February), Denmark (April), Norway (April), China (May), France (May) and Germany (May). We are committed to reviewing the literature on this topic regularly as new data emerge and to re-evaluate the conclusions of this review given the rapid pace of ongoing research on COVID-19. These updates will be available online (https://www.ed.ac.uk/usher/uncover).

Children are less likely to suffer severe and critical COVID-19 disease than adults [24], however, a new paediatric multisystem inflammatory syndrome associated with SARS-CoV-2 epidemic has recently been identified in the UK, the US, France, Italy, Spain and Switzerland. A small number of children with diagnosed COVID-19 developed a significant systemic inflammatory response with a higher rate of cardiac involvement, caused by a severe form of Kawasaki-like disease [25]. This condition could be fatal and requires prompt and aggressive medical interventions. This has triggered the release of a new guidance from the Royal College of Paediatrics and Child Health [26]. Research on this topic is currently ongoing (DIAMONDS, ISARIC) and a new study from the British Paediatric Surveillance Unit will be launching soon.

This rapid review has a number of limitations. Despite experienced reviewers undertaking searches, screening and data extraction, due to the tight deadline the literature review and data extraction were done by one person per article so it is possible that some key articles may have been missed. We have not performed quality assessment for the included studies, so this may have biased the results. Many of the included studies are pre-print publications or reports and therefore not peer-reviewed. This review should not replace individual clinical judgement and the sources cited should be checked. The views expressed represent those of the authors and are not a substitute for professional medical advice.

## CONCLUSIONS

There is very limited evidence on paediatric cases acting as a source of infection, which highlights the importance of obtaining robust data on transmission dynamics in children in future studies. Preliminary results from large targeted, population and school studies suggest that children may be less likely to be infected or infect others. Further seroprevalence studies (powered adequately for the paediatric population), are urgently required to establish whether children are in fact less likely to be infected compared to adults. Faeco-oral transmission appears to pose a higher risk of onward transmission in infected children compared to adults, given the longer faecal shedding time observed in several studies. This may have substantial implications for community spread in day-care centres, schools, and homes and the need for a high level of basic hygiene measures in these settings to limit spread.

## Additional material

Online Supplementary Document
