# Supplementary material for: The role of children in transmission of SARS-CoV-2: A rapid review
Source: J Glob Health. 2020 Jun 29;10(1):011101. doi: 10.7189/jogh.10.011101 (PMC7323934; doi:10.7189/jogh.10.011101)
Supplement: Online Supplementary Document [file jogh-10-011101-s001.pdf]

Appendix:

Pubmed

Search date: 2020-04-30

414 results

(Schools[mh:noexp] OR schools, nursery[mh] OR “Child Day Care Centers”[mh] OR “Nurseries, Infant”[mh] OR school[tiab] OR schools[tiab] OR preschools[tiab] OR preschool[tiab] OR “pre school”[tiab] OR “pre schools”[tiab] OR nursery[tiab] OR nurseries[tiab] OR kindergarten[tiab] OR ((“day care” OR daycare) AND (child* OR infant*)) OR pediatric[tiab] OR paediatric[tiab] OR child[tiab] or children[tiab] or schoolchild*[tiab] OR young*[tiab] OR teen*[tiab] OR adolescen*

AND

(("Betacoronavirus"[Mesh] OR "Coronavirus Infections"[MH] OR "Spike Glycoprotein, COVID-19 Virus”[NM] OR "COVID-19"[NM] OR "Coronavirus"[MH] OR "Severe Acute Respiratory Syndrome Coronavirus 2"[NM] OR 2019nCoV[ALL] OR Betacoronavirus*[ALL] OR Corona Virus*[ALL] OR Coronavirus*[ALL] OR Coronovirus*[ALL] OR CoV[ALL] OR CoV2[ALL] OR COVID[ALL] OR COVID19[ALL] OR COVID-19[ALL] OR HCoV-19[ALL] OR nCoV[ALL] OR "SARS CoV 2"[ALL] OR SARS2[ALL] OR SARSCoV[ALL] OR SARS-CoV[ALL] OR SARS-CoV-2[ALL] OR Severe Acute Respiratory Syndrome CoV*[ALL])

AND

(((2020/04/01[EDAT] : 3000[EDAT]) OR (2020/04/01[PDAT] : 3000[PDAT]))))

WHO COVID-19 database

https://search.bvsalud.org/global-literature-on-novel-coronavirus-2019-ncov/

Search date: 2020-04-30

531 results

(tw:(child* OR infan* OR baby OR babies OR pediatric OR paediatric OR young* OR teen* OR adolescen* OR school* OR nurser* OR preschool* OR pre-school* OR kindergarten*)) AND (entry_date:202004*)

medRxiv via https://mcguinlu.shinyapps.io/medrxivr/

Search date 2020-04-30

127 results

Covid-19 cluster combined with OR:

COVID-19

[Cc]oronavirus

SARS-CoV-2

2019-nCoV

Population cluster combined with OR:

[Cc]hild

[Ii]nfan

[Bb]aby

[Bb]abies

[Pp]ediatric

[Pp]aediatric

[Yy]oung

[Tt]een

[Aa]dolescen

[Ss]chool

[Nn]urser

[Pp]reschool

[Pp]re-school

[Kk]indergarten
